# Proanthocyanidin to prevent formation of the reexpansion pulmonary edema

**DOI:** 10.1186/1749-8090-4-40

**Published:** 2009-07-28

**Authors:** Orhan Yucel, Ergun Ucar, Ergun Tozkoparan, Armagan Gunal, Cemal Akay, Mehmet Ali Sahin, Onur Genc

**Affiliations:** 1 Department of Thoracic Surgery, Gulhane Military Medical Academy, Ankara, Turkey; 2 Department of Pulmonary Medicine, Gulhane Military Medical Academy, Ankara, Turkey; 3 Department of Pathology, Gulhane Military Medical Academy, Ankara, Turkey; 4 Department of Pharmaceutical Toxicology, Gulhane Military Medical Academy, Ankara, Turkey; 5 Department of Cardiovascular Surgery, Gulhane Military Medical Academy, Ankara, Turkey

## Abstract

**Background:**

We aimed to investigate the preventive effect of Proanthocyanidine (PC) in the prevention of RPE formation.

**Methods:**

Subjects were divided into four groups each containing 10 rats. In the Control Group (CG): RPE wasn't performed. Then subjects were followed up for three days and they were sacrificed after the follow up period. Samplings were made from tissues for measurement of biochemical and histopathologic parameters. In the Second Group (PCG): The same protocol as CG was applied, except the administration of PC to the subjects. In the third RPE Group (RPEG): Again the same protocol as CG was applied, but as a difference, RPE was performed. In the Treatment Group (TG): The same protocol as RPEG was applied except the administration of PC to the subjects.

**Results:**

In RPEG group, the most important histopathological finding was severe pulmonary edema with alveolar damage and acute inflammatory cells. These findings were less in the TG group. RPE caused increased MDA levels, and decreased GPx, SOD and CAT activity significantly in lung tissue.

**Conclusion:**

PC decreased MDA levels. Oxidative stress plays an important role in pathophysiology of RPE and PC treatment was shown to be useful to prevent formation of RPE.

## Introduction

Reexpansion pulmonary edema (RPE) is a rare and acute rare complication, occurring after rapid reinflation of a collapsed lung, generally encountered after evacuation of large amount of air or fluid from the pleural space [1]. The potentially lethal complication of RPE is unilateral lung injury, which is initiated by cytotoxic oxygen metabolites and associated with a temporarily influx of polymorphonuclear neutrophils [1]. These toxic oxygen metabolites may occur as a result of reoxygenation of a collapsed lung.

Proanthocyanidine (PC) is a combination of biologically active polyphenolic flavonoids. They include oligomeric PC, and they have been demonstrated to exert a novel spectrum of biological, pharmacological, therapeutic, and chemoprotective properties against oxidative stress and oxygen free radicals [2,3]. PC manifests its novel mechanistic pathways of cardioprotection by potent hydroxyl and other free radical scavenging abilities [4,5]. Recently it has been emphasized that, as compared to Vitamins C, E and β-carotene, PC provides better antioxidant efficacy [4]. However Pataki et al., (2001) reported that PC improves cardiac recovery during reperfusion of ischemic conditions [5]. Based on the preventive effect of PC in this experimental research, we aimed to investigate the possible beneficial protective effects of PC in RPE.

## Methods

The study was performed in Animal Research Laboratory. Institutional ethic committee permission was obtained before the study. Forty adult Rates Norvecus weighing between 150 and 170 grams were used. A commercially available PC was obtained from GNC Bakara LTD. (PC: 100 mg, 90 capsules, Istanbul, TR).

In this experimental study, forty rats were separated into four groups by the simple random sampling method with each group containing ten rats.

The first group was the Control Group (CG). In this group, no Pneumothorax (Px) and subsequent RPE was performed. Subjects have been followed for three days. In CG, 2 ml of 1% methylcellulose solution diluted with 0.9% NaCl to 10 ml was given for 3 days by gavage. After the follow up period, the rats were sacrificed. Then samplings from the tissues have been carried out for measurement of histopathological and biochemical parameters (superoxide dismutase (SOD), glutathion peroxidase (GPx), catalase (CAT), malondialdehyde (MDA)) and the results were recorded.

The second group was PC Group (PCG). The same protocol with CG (three days of follow up, sacrification, tissue sampling for histopathological and biochemical analysis, recording of results) were applied. The only difference from CG was the administration of PC (100 mg/kg/day), by gavage, during the 3 day follow up period. Before the administration of PC, it was homogenized in 2 ml, 1% methylcellulose solution and then diluted with 0.9% NaCl to 10 ml [6].

The third group was RPE Group (RPEG). The same protocols with the CG (three days of follow up, sacrification, tissue sampling for histopathological and biochemical analysis, and recording of results) have been applied. The only difference from CG group was the performance of RPE. The RPE forming protocol is summarized below (*). Two hours after re-expansion, all rats were sacrificed and tissue samples were taken.

The fourth group was the Treatment Group (TG); which was designed like the combination of RPE and PC groups. In this group, the same protocol with RPEG (Px and RPE formation, sacrification, tissue sampling for histopathological and biochemical analysis, and data recording) was applied, except the administration of PC, which was started 8 h before Px application, and continued for 72 hours with the same daily doze and route as PCG. This is summarized in Table 1.

**Table 1:** Special features in our experimental study groups are listed below.

Group	(n)	Nourishment	Follow Up (Day)	RPE procedure (+/-)
CG	10	Rat food	3	-
PCG	10	Rat food and PC	3	-
RPEG	10	Rat food	3	+
TG	10	Rat food and PC	3	+

CG; Control Group, PCG: Proanthocyanidine Group, RPEG; Reexpansion Pulmonary Edema, Group. TG: Treatment, Reexpansion Pulmonary Edema Plus Proanthocyanidin, Group. Rat food: All animals were provided access to the same food (2630 kkal/kg metabolic energy, 21% protein, 7% cellulose, and fat 9%) for a period of 7 days. PC: In PCG, PC (100+/-5 mg/kg rat body weight) intake was performed seven days by gavage orally. In TG, PC intake was started 8 h before pneumothorax application and continued 72 hour by the same doze and way of PCG. RPE procedure: This column shows whether reexpansion pulmonary edema was performed or not.

### (*) RPE Forming Protocol

**Figure 1:**
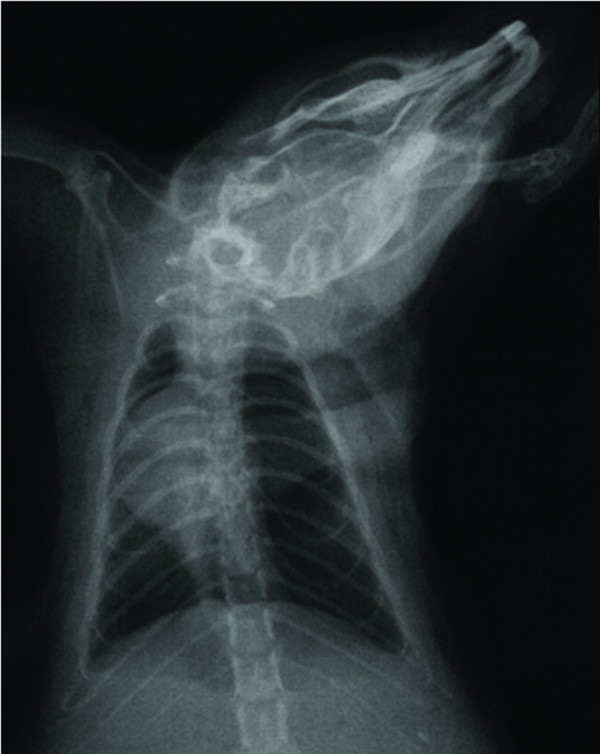
**The confirmation of right pneumothorax by chest X-ray**.

**Figure 2:**
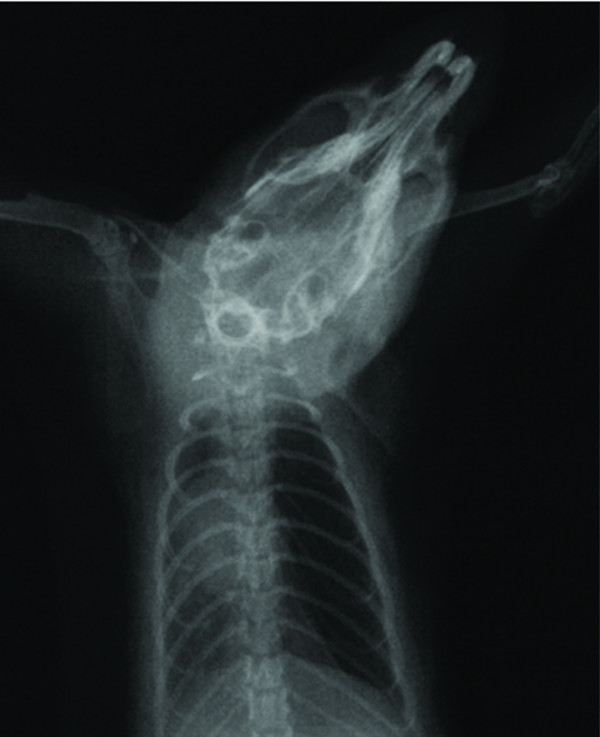
**The confirmation of right re-expansion by chest X-ray**. Re-expansion pulmonary edema is also seen.

**Figure 3:**
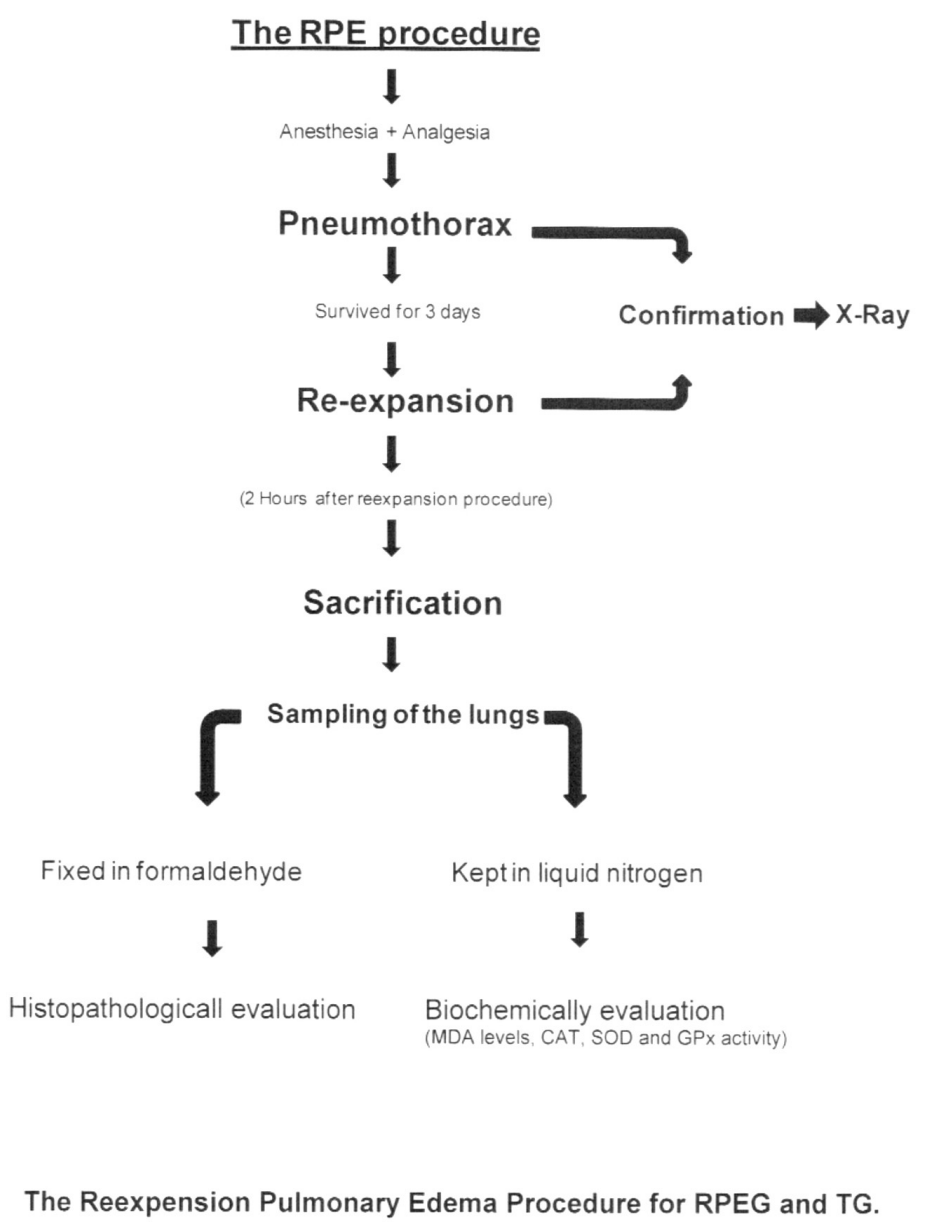
**The Reexpension Pulmonary Edema Procedure for RPEG and TG**.

We used the same RPE forming model, as our previous study [1]. Briefly, rats were anesthetized with intraperitoneal Ketamine Hydrocloride (Ketamine hydrochloride solution in % 5, Parke – Davis license Eczacıbaşı; Medical Industry, Istanbul) 90 mg/kg and Xylazine (Xylazine solution in % 2, by Parke – Davis license Eczacıbaşı Medical Industry, Istanbul) 10 mg/kg. In RPEG and RPE + PCG, pneumothorax was induced by injecting about 4 ml of air into the thorax via percutaneous route with a 22 gauge cannula which was placed in the right hemithorax. The adequacy of the pneumothorax was confirmed with control X-rays in all rats (Figure 1). Thereafter, the animals were allowed to survive for an additional 72 h. Then, in both RPEG and TG, pneumothorax was treated by aspiration of the air, quickly with a 22 gauge cannula. The adequacy of the reexpansion was confirmed with control X-rays (Figure 2) and also during sternotomy in all rats. Two hours after reexpansion, all rats were sacrificed by giving lethal dose of Xylazine and Ketamine. Their chests were opened by median sternotomy, and their lungs were removed immediately for histopathological and biochemical sampling. For histopathological assessment, lungs were filled with 10% buffered formalin solution via intratracheal instillation, and were fixed in the same solution. Before fixation, one third of upper lobes of both lungs were kept in liquid nitrogen for analysis of oxidative stress. The RPE procedure is summarized in Figure 3.

### Tissue preparation for histopathological evaluation

Lung samples were embedded in paraffin blocks. Four μm sections were sliced from paraffin blocks and stained with hematoxylin-eosin (HE). Pulmonary edema was evaluated by a pathologist, who was blinded to groups. We have developed a "pulmonary edema score" (PES) in order to assess the degree of pulmonary edema. Briefly, each animal was classified according to the presence of above mentioned histopathological findings as minimal, moderate and advanced. Minimal pulmonary edema (score 1); included those with only fluid extravasations, moderate edema (score 2); included those with fluid extravasations and fluid in the alveoli, advanced edema (score 3); included animals that have typical histopathological findings of pulmonary edema, and eventually those with normal pulmonary parenchyma were classified as score 0.

### Analysis of Parameters Related to Oxidative Stress Status

Malondialdehyde (MDA) levels, Catalase (CAT), Superoxide Dismutase (SOD) and Glutathione Peroxidase (GPx) activity in tissue homogenate samples were measured in accordance with the method described in our previous study [1]. Tissue preparation for oxidative stress status: Tissue samples were homogenized with ice-cold KCl (1.15 %) using a glass homogenizer. The homogenates was then centrifuged at 4400 g for 10 min at 4°C to remove the cell debris and the obtained supernatant was used for the determination of MDA and antioxidant enzymes. GPx activity measurement: The reaction mixture was 50 mMol tris buffer with pH 7.6; containing 1 mMol of Na_2 _EDTA, 2 mMol of reduced glutathione (GSH), 0.2 mMol of NADPH, 4 mMol of sodium azide and 1000 U of glutathione reductase (GR). 50 μL of plasma or tissue homogenate and 950 μL of reaction mixture were mixed and incubated for 5 min. at 37°C. Then the reaction was initiated with 10 μL of t-butyl hydroperoxide (8 mMol) and the decrease in NADPH absorbance was followed at 340 nm for 3 min. Enzyme activities were reported as U/g in tissue. MDA level measurement: MDA levels were expressed as TBARS. After the reaction of thiobarbituric acid with MDA, the reaction product was measured spectrophotometrically. Tetramethoxy propane solution was used as a standard. SOD activity measurement: Each homogenate was diluted 1:400 with 10 mM phosphate buffer, pH 7.00. 25 μL of diluted hemolysate was mixed with 850 μL of substrate solution containing 0.05 mMol xanthine sodium and 0.025 mmol/L 2-(4-iodophenyl)-3-(4-nitrophenol)-5- phenyltetrazolium chloride (INT) in a buffer solution containing 50 mMol CAPS and 0.94 mMol EDTA pH 10.2. Then, 125 μL of xanthine oxidase (80 U/L) was added to the mixture and absorbance increase was followed at 505 nm for 3 minutes against air. 25 μL of phosphate buffer or 25 μL of various standard concentrations in place of sample were used as blank or standard determinations. CuZn-SOD activity was expressed in U/g tissue. CAT activity measurement: The reaction mixture was 50 mMol phosphate buffer pH 7.0, 10 mMol H_2_O_2 _and homogenate. The reduction rate of H_2_O_2 _was followed at 240 nm for 30 seconds at room temperature. Catalase activity was expressed in KU/g tissue.

### Statistical analysis

Statistical analysis was done to analyze each group mutually by using Kruskal-Wallis and Bonferroni-corrected Mann-Whitney U tests. The histopathological and biochemical results were expressed as the standard deviation (min-max) and p < 0.05 was assessed as statistically significant.

## Results

### Histopathological evaluation

**Figure 4:**
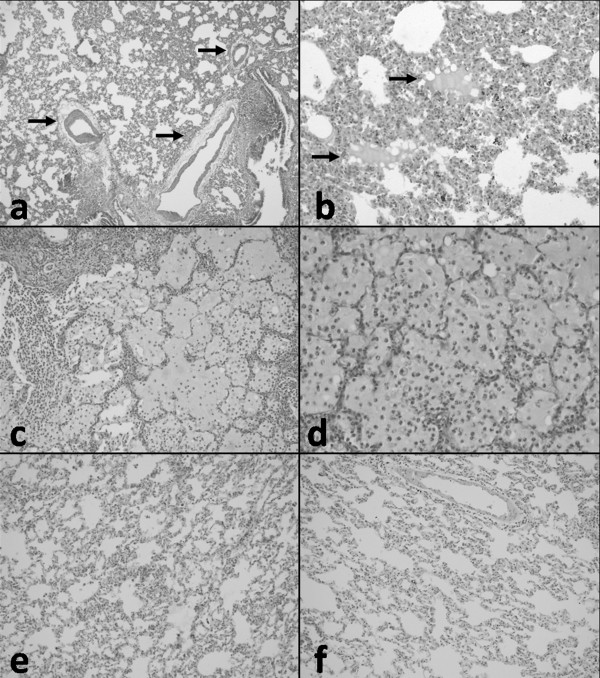
**(a) Fluid extravasation in the perivascular areas (arrows) in TG (HE × 100), (b) Eosinophilic fluid accumulation in some of the alveolar spaces (arrows) in TG (HE × 200), (c) Severe pulmonary edema in RPEG, (HE × 100), (d) with alveolar damage and scattered acute inflammatory cells, typical RPE, in RPEG (HE × 200)**. **(e) **Normal pulmoner histological structures in PCG (HE × 200) and **(f) **in CG also seen HE × 200).

Histological parameters included normal pulmonary parenchyma, fluid extravasations, fluid extravasations and fluid in the alveoli, and typical pulmonary edema. The fluid accumulation in alveoli and extravasation of fluid were the most common findings in histopathological examination, and these two findings represented pulmonary edema. In TG, the most common findings were fluid extravasation in the perivascular areas (Figure 4a) and eosinophilic fluid accumulation in some of the alveolar spaces (Figure 4b). In RPEG, the most important histopathological finding was severe pulmonary edema (Figure 4c) with alveolar damage and scattered acute inflammatory cells (Figure 4d), typical for RPE. We showed that histopathological findings of normal PCG and CG are alike (Figure 4e, 4f). No pathologic findings were noted during the histopathological evaluation of CG rats' lung tissues (Figure 4f). TG had statistically significant lower mean PES (1.00 ± 0.82) with respect to RPEG (2,10 ± 2,74; p = 0,011). The fluid accumulations in alveoli and extra vascular area were significantly less in the TG. Morphologic patterns of those obtained from sections were summarized in Table 2.

**Table 2:** Histopathologic results of lung tissue.

Histological findings	CG n = 10	PCG n = 10	RPEG n = 10	TG n = 10
Normal pulmonary parenchyma (edema score 0)	10	10	0	3
Fluid extravasations (edema score 1)	0	0	2	4
Fluid extravasations and fluid in the alveoli (edema score 2)	0	0	5	3
Pulmonary edema (edema score 3)	0	0	3	0
Mean pulmonary edema score	0	0	2,10 ± 2,74*	1,00 ± 0,82*

* p = 0,011

CG; Control Group, PCG: Proanthocyanidine Group, RPEG; Reexpansion Pulmonary Edema Group, TG: Treatment, Reexpansion Pulmonary Edema Plus Proanthocyanidin, Group.

### GPx, SOD and CAT activities, and MDA levels in lung tissue

Oxidative stress status analysis included SOD, CAT and GPx activity, and MDA levels. RPE caused significantly increased MDA levels, and decreased GPx, SOD and CAT activity in lung tissue. PC treatment decreased MDA levels, but SOD, CAT and GPx activities were similar to those of RPEG. MDA levels and GPx, SOD and CAT activities in lung tissue are presented in Table 3.

**Table 3:** Oxidative stress related parameters of the lung tissue.

**Parameters**	**CG**	**PCG**	**RPEG**	**TG**	**Significance**			
					
					**CG-PCG**	**CG-RPEG**	**CG- TG**	**RPEG- RPE + PC**
MDA (nmol/g)	6.56 ± 0.15	6.45 ± 0.12	7.47 ± 0.17	7.03 ± 0.29	NS	p < 0.001	p = 0.002	p < 0.001

GPx (U/g)	48.24 ± 2.97	48.21 ± 2.78	35.12 ± 2.54	38.21 ± 4.53	NS	p < 0.001	P < 0.001	NS

CAT U/g)	3.27 ± 0.22	3.31 ± 0.24	3.01 ± 0.48	3.05 ± 0.74	NS	NS	NS	NS

SOD (U/g)	255.31 ± 13.45	265.31 ± 11.42	109.23 ± 4.34	134.25 ± 19.73	NS	p < 0.001	P < 0.001	p = 0.002

CG; Control Group, PCG: Proanthocyanidine Group, RPEG; Reexpansion Pulmonary Edema Group, TG: Reexpansion Pulmonary Edema Plus Proanthocyanidin Group. MDA; Malondialdehyde, CAT; Catalase, SOD; Superoxide Dismutase, GPx; Glutathione Peroxides. NS: Not Significant.

## Discussion

In the current study we have demonstrated that; in an animal model of RPE, malondialdehyde (MDA) level of pulmonary parenchymal tissue, a marker of oxidative stress, increased and antioxidant enzyme activities of GPx and SOD decreased. Treatment with PC partially improved decreased SOD and GPx activities, and decreased MDA levels. PC treatment also resulted in less severe pulmonary edema in rats with RPE. In the process of RPE, two main contributing factors are; the amount of drained fluid or air, and the chronicity of the lung collapse. There are other minor contributing factors such as; reexpansion technique, pulmonary arterial hypertension, associated hypoxemia and bronchial obstruction [7]. A lung collapse longer than 72 hours and rapid evacuation of the fluid or air from the pleural space leading to an end-expiratory pleural pressure less than -20 cm H_2_O, is associated with higher risk of RPE [7]. However, exact mechanisms in pathophysiology of RPE have not been fully understood yet. Recent studies have demonstrated that several mechanisms; such as excessive negative pressure, increase in pulmonary vascular permeability and capillary pressure of the lung, mechanical damage of alveoli due to abrupt distension, loss of surfactant, migration of inflammatory cells, release of inflammatory mediators, increase of cytokines and free radicals probably due to hypoxic injury of the atelectatic lung may be involved in pathogenesis of RPE [6,8-11]. More than one century ago, Reisman and Hartkey used the terms of "albuminous expectoration" and "albumin sputum" in cases that developed pulmonary edema after removal of a large amount of pleural fluid [7,12]. These observations have been the first data, explaining mechanism of RPE, which reflect marked increase in lung microvascular permeability. The alteration of microvascular permeability may be due to two main causes; one of them is mechanical destruction of alveolar wall by abrupt distension [7], and second mechanism, probably more dominant, is ischemic-reperfusion injury, which may occur in many other organs [[10,11], and [12]]. During reperfusion of the lung, free radicals, lipid and polypeptide mediators increase, which cause the endothelium to damage, with a subsequent increase in vascular permeability [11,12]. A study evaluating edematous fluids in two patients with RPE reported the fluid/plasma ratio of total protein concentration to be higher than 0.7, indicating an increase in vascular permeability and this result has also been confirmed by the increase in polymorphonuclear leukocytes (PMNL) and some arachidonic acid metabolites [9]. They have also suggested that re-expansion of the collapsed lung causes acute inflammation in the lungs, and PMNLs play an important role in the mechanism of the increase in pulmonary microvascular permeability. An animal study has shown that PMNLs and pro-inflammatory cytokines, interleukin (IL) 8 and monocyte chemoattractant protein 1, are involved in the development of RPE [13,14]. Furthermore, some studies have shown that; hypoxia-reoxygenation injury of one lung may cause acute respiratory distress syndrome (ARDS) in the other, along with systemic multi-organ injuries [15]. According to a study it is suggested that; pathophysiology of RPE was very similar to that of ARDS, since both were characterized by intra-alveolar activated PMNLs and markedly increased lung microvascular permeability [12]. Reactive oxygen species might also have a role in the development of RPE, probably by causing PMNL influx to the lungs and causing endothelial damage [16,17]. A study group reported that; reexpansion of the collapsed lung with air causes marked PMNL accumulation and reactive oxygen species (ROS) production, and the latter was minimal in case of reexpansion of the lungs with nitrogen [16]. It was also reported that; activation of sequestered PMNLs in the pulmonary circulation caused the release of ROS [18]. These data indicate that an inflammatory process, in which oxidative stress is involved, play a key role in the increase of lung capillary permeability and the consequent development of RPE. MDA is a lipid peroxidation product and frequently used as a marker of oxidative stress [19]. SOD, which functions as the primary enzymatic defense against superoxide radicals, catalase and GPx both of which decompose hydrogen peroxide to form water and oxygen, are the most commonly examined antioxidant enzymes [19]. The decrease in these antioxidant enzyme activities indicate oxidant/antioxidant imbalance in favor of oxidants, i.e. oxidative stress. In our animal model of RPE, increase in MDA and decrease in SOD and GPx supports, from another point of view, the suggestion that oxidative stress play a key role in RPE pathophysiology. PC is oligomeric and polymeric end products of the flavonoid biosynthesis pathway, and is present in fruits, bark, leave and seeds of many plants and grape seeds as well [2,3]. PC has antibacterial, antiviral, anticarcigogenic, anti-inflammatory, anti-allergic, vasodilator and free radical scavenging activities [2-4]. More importantly, regarding the pathophysiology of RPE, it has also been shown to inhibit lipid peroxidation, capillary permeability, inflammatory enzymes of arachidonic acid metabolism and formation of IL-6 and IL-8, latter of which involve in RPE development [[2,20], and [21]]. Moreover, in vitro studies of PC extract demonstrated better anti oxidant activity than vitamin C, vitamin E and beta carotene and their combinations [21-23]. PC preferentially binds to areas with high glycosaminoglycan content, such as capillary wall and consequently decreases vascular permeability, enhances capillary strength and vascular function [2]. These data might be the explanation of finding that PC treatment leads to less pulmonary edema in the animal model of RPE. Despite the beneficial effects of PC as mentioned above, we had some doubts about the possible harmful effects of it on lung tissue. For this reason, we formed another group, namely PCG, which we only administrated PC. At the end, by comparing the histopathological findings of PCG and CG, no significant evidence have been found supporting any kind of harmful effect of PC on lung tissue. In conclusion, we have suggested that oxidative stress involves in pathophysiology of RPE, and PC treatment may prevent formation of RPE partially, or decrease the intensity of RPE by its antioxidant activity. Our point was to show the beneficial effects of PC in RPE, like protection and prevention, therefore, we used a single dosage and time schedule. In order to answer various questions that can be asked about the more effective usage of the molecule, we need to set further experiments related to some factors like proper treatment dosage, suitable way of administration, and most appropriate interval of treatment.

## Competing interests

The authors declare that they have no competing interests.

## Authors' contributions

OY and EU were involved with study design, performed the data analysis and all the OY, MAS and EU were involved with study design, performed the data analysis and all the operations. CA was designed the study and performed data analysis. ET did the background literature search. The lung samples were evaluated by AG. OG was designed the study and has given final approval of the version to be published. All authors have read and approved the manuscript.
